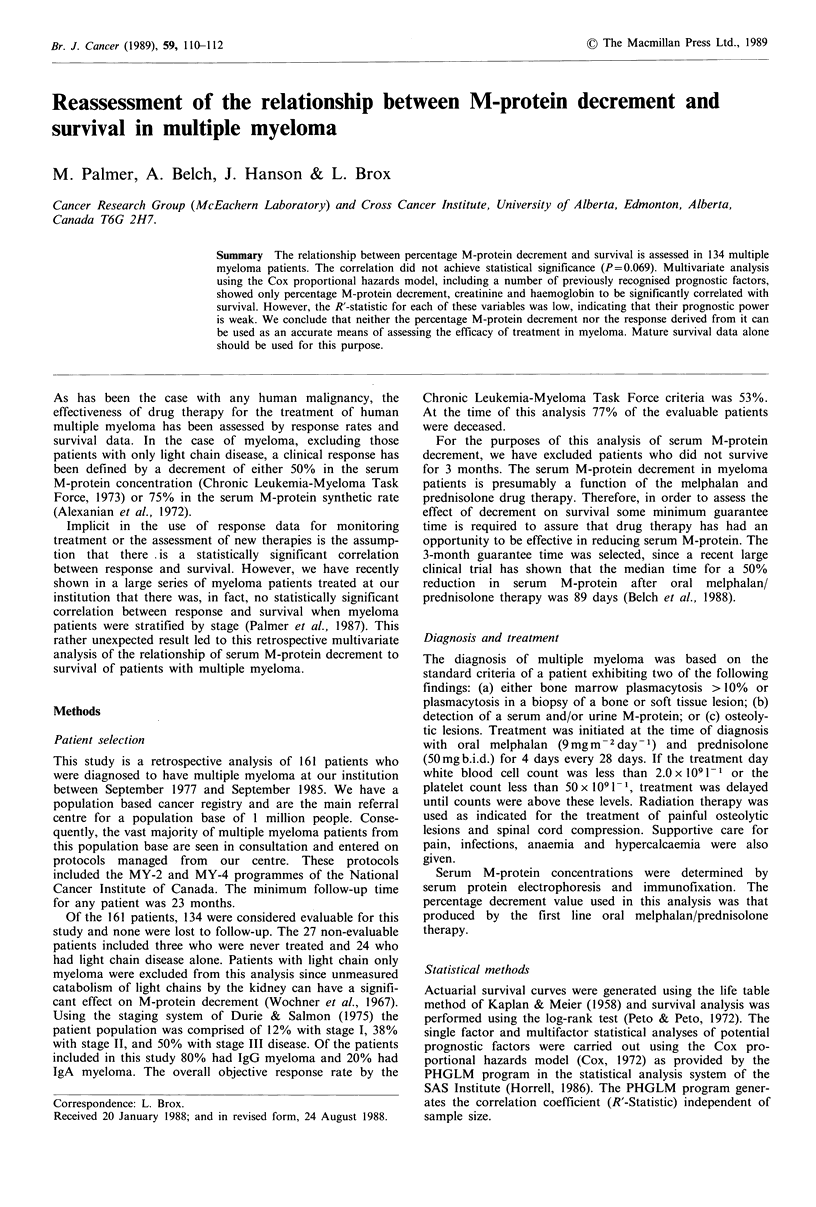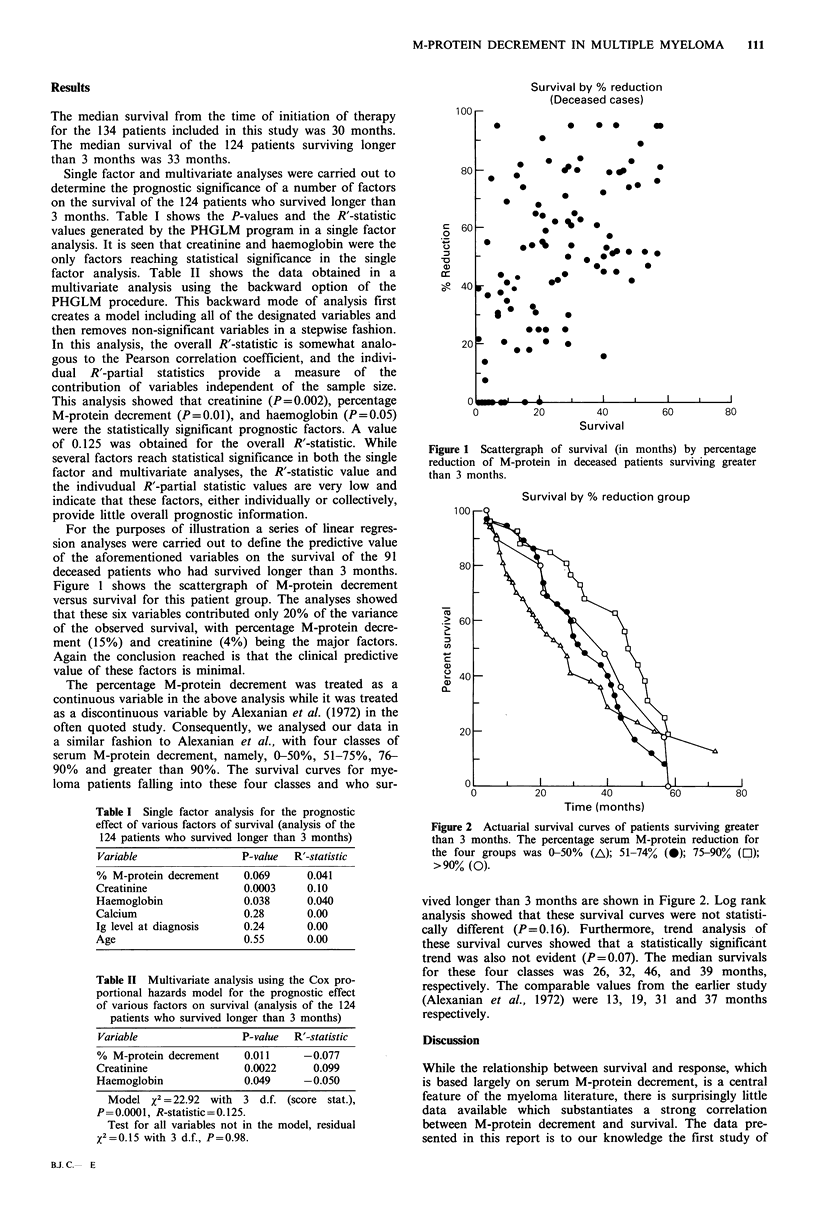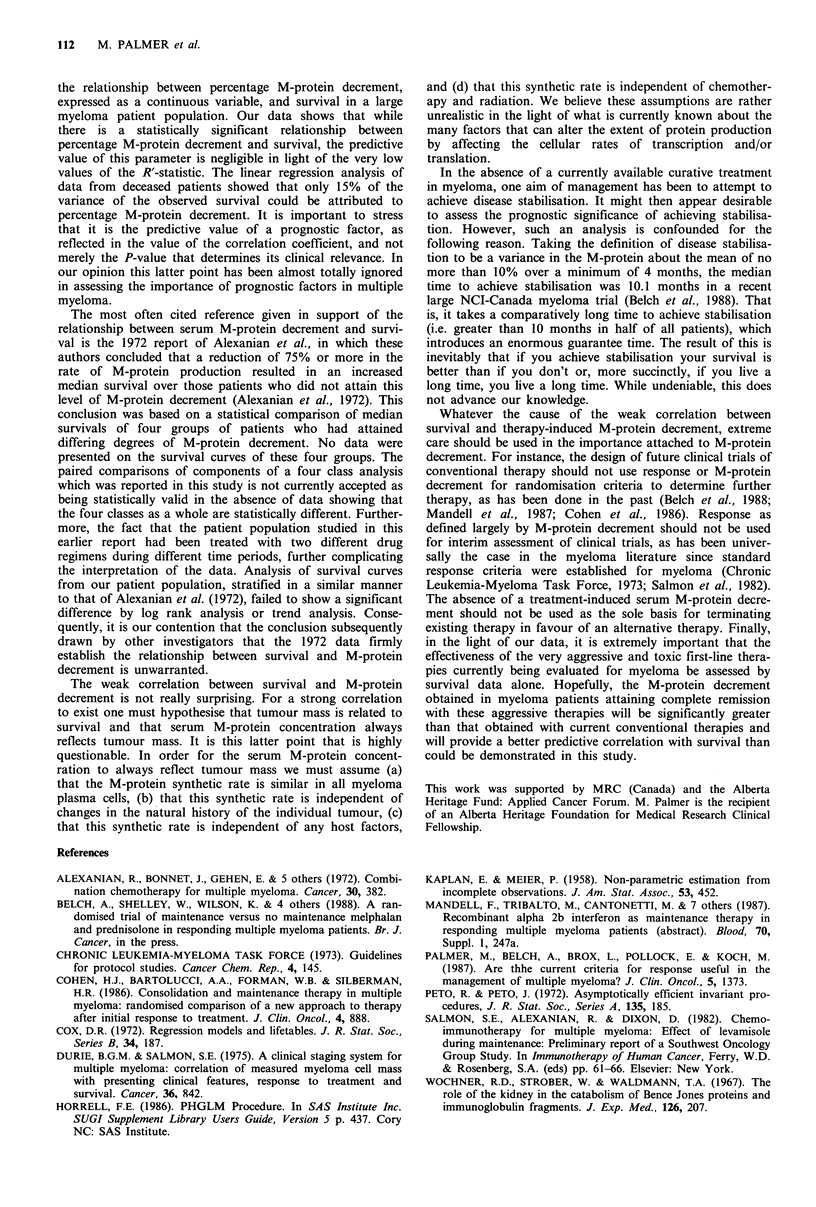# Reassessment of the relationship between M-protein decrement and survival in multiple myeloma.

**DOI:** 10.1038/bjc.1989.22

**Published:** 1989-01

**Authors:** M. Palmer, A. Belch, J. Hanson, L. Brox

**Affiliations:** Cancer Research Group, University of Alberta, Edmonton, Alberta, Canada.

## Abstract

The relationship between percentage M-protein decrement and survival is assessed in 134 multiple myeloma patients. The correlation did not achieve statistical significance (P = 0.069). Multivariate analysis using the Cox proportional hazards model, including a number of previously recognised prognostic factors, showed only percentage M-protein decrement, creatinine and haemoglobin to be significantly correlated with survival. However, the R'-statistic for each of these variables was low, indicating that their prognostic power is weak. We conclude that neither the percentage M-protein decrement nor the response derived from it can be used as an accurate means of assessing the efficacy of treatment in myeloma. Mature survival data alone should be used for this purpose.


Br. J. Cancer (1989), 59, 110–112

© The Macmillan Press Ltd., 1989

### Reassessment of the relationship between M-protein decrement and survival in multiple myeloma

M. Palmer, A. Belch, J. Hanson & L. Brox

*Cancer Research Group (McEachern Laboratory) and Cross Cancer Institute, University of Alberta, Edmonton, Alberta, Canada T6G 2H7.*

**Summary** The relationship between percentage M-protein decrement and survival is assessed in 134 multiple myeloma patients. The correlation did not achieve statistical significance ($P=0.069$). Multivariate analysis using the Cox proportional hazards model, including a number of previously recognised prognostic factors, showed only percentage M-protein decrement, creatinine and haemoglobin to be significantly correlated with survival. However, the $R'$-statistic for each of these variables was low, indicating that their prognostic power is weak. We conclude that neither the percentage M-protein decrement nor the response derived from it can be used as an accurate means of assessing the efficacy of treatment in myeloma. Mature survival data alone should be used for this purpose.

As has been the case with any human malignancy, the effectiveness of drug therapy for the treatment of human multiple myeloma has been assessed by response rates and survival data. In the case of myeloma, excluding those patients with only light chain disease, a clinical response has been defined by a decrement of either 50% in the serum M-protein concentration (Chronic Leukemia-Myeloma Task Force, 1973) or 75% in the serum M-protein synthetic rate (Alexanian *et al.*, 1972).

Implicit in the use of response data for monitoring treatment or the assessment of new therapies is the assumption that there is a statistically significant correlation between response and survival. However, we have recently shown in a large series of myeloma patients treated at our institution that there was, in fact, no statistically significant correlation between response and survival when myeloma patients were stratified by stage (Palmer *et al.*, 1987). This rather unexpected result led to this retrospective multivariate analysis of the relationship of serum M-protein decrement to survival of patients with multiple myeloma.

### Methods

### *Patient selection*

This study is a retrospective analysis of 161 patients who were diagnosed to have multiple myeloma at our institution between September 1977 and September 1985. We have a population based cancer registry and are the main referral centre for a population base of 1 million people. Consequently, the vast majority of multiple myeloma patients from this population base are seen in consultation and entered on protocols managed from our centre. These protocols included the MY-2 and MY-4 programmes of the National Cancer Institute of Canada. The minimum follow-up time for any patient was 23 months.

Of the 161 patients, 134 were considered evaluable for this study and none were lost to follow-up. The 27 non-evaluable patients included three who were never treated and 24 who had light chain disease alone. Patients with light chain only myeloma were excluded from this analysis since unmeasured catabolism of light chains by the kidney can have a significant effect on M-protein decrement (Wochner *et al.*, 1967). Using the staging system of Durie & Salmon (1975) the patient population was comprised of 12% with stage I, 38% with stage II, and 50% with stage III disease. Of the patients included in this study 80% had IgG myeloma and 20% had IgA myeloma. The overall objective response rate by the Chronic Leukemia-Myeloma Task Force criteria was 53%. At the time of this analysis 77% of the evaluable patients were deceased.

For the purposes of this analysis of serum M-protein decrement, we have excluded patients who did not survive for 3 months. The serum M-protein decrement in myeloma patients is presumably a function of the melphalan and prednisolone drug therapy. Therefore, in order to assess the effect of decrement on survival some minimum guarantee time is required to assure that drug therapy has had an opportunity to be effective in reducing serum M-protein. The 3-month guarantee time was selected, since a recent large clinical trial has shown that the median time for a 50% reduction in serum M-protein after oral melphalan/prednisolone therapy was 89 days (Belch *et al.*, 1988).

### *Diagnosis and treatment*

The diagnosis of multiple myeloma was based on the standard criteria of a patient exhibiting two of the following findings: (a) either bone marrow plasmacytosis >10% or plasmacytosis in a biopsy of a bone or soft tissue lesion; (b) detection of a serum and/or urine M-protein; or (c) osteolytic lesions. Treatment was initiated at the time of diagnosis with oral melphalan ($9\,mg\,m^{-2}\,day^{-1}$) and prednisolone (50 mg b.i.d.) for 4 days every 28 days. If the treatment day white blood cell count was less than $2.0\times10^{9}\,l^{-1}$ or the platelet count less than $50\times10^{9}\,l^{-1}$, treatment was delayed until counts were above these levels. Radiation therapy was used as indicated for the treatment of painful osteolytic lesions and spinal cord compression. Supportive care for pain, infections, anaemia and hypercalcaemia were also given.

Serum M-protein concentrations were determined by serum protein electrophoresis and immunofixation. The percentage decrement value used in this analysis was that produced by the first line oral melphalan/prednisolone therapy.

### *Statistical methods*

Actuarial survival curves were generated using the life table method of Kaplan & Meier (1958) and survival analysis was performed using the log-rank test (Peto & Peto, 1972). The single factor and multifactor statistical analyses of potential prognostic factors were carried out using the Cox proportional hazards model (Cox, 1972) as provided by the PHGLM program in the statistical analysis system of the SAS Institute (Horrell, 1986). The PHGLM program generates the correlation coefficient ($R'$-Statistic) independent of sample size.

Correspondence: L. Brox.

Received 20 January 1988; and in revised form, 24 August 1988.

### Results

The median survival from the time of initiation of therapy for the 134 patients included in this study was 30 months. The median survival of the 124 patients surviving longer than 3 months was 33 months.

Single factor and multivariate analyses were carried out to determine the prognostic significance of a number of factors on the survival of the 124 patients who survived longer than 3 months. Table I shows the *P*-values and the *R'*-statistic values generated by the PHGLM program in a single factor analysis. It is seen that creatinine and haemoglobin were the only factors reaching statistical significance in the single factor analysis. Table II shows the data obtained in a multivariate analysis using the backward option of the PHGLM procedure. This backward mode of analysis first creates a model including all of the designated variables and then removes non-significant variables in a stepwise fashion. In this analysis, the overall *R'*-statistic is somewhat analogous to the Pearson correlation coefficient, and the individual *R'*-partial statistics provide a measure of the contribution of variables independent of the sample size. This analysis showed that creatinine ($P=0.002$), percentage M-protein decrement ($P=0.01$), and haemoglobin ($P=0.05$) were the statistically significant prognostic factors. A value of 0.125 was obtained for the overall *R'*-statistic. While several factors reach statistical significance in both the single factor and multivariate analyses, the *R'*-statistic value and the indivudual *R'*-partial statistic values are very low and indicate that these factors, either individually or collectively, provide little overall prognostic information.

For the purposes of illustration a series of linear regression analyses were carried out to define the predictive value of the aforementioned variables on the survival of the 91 deceased patients who had survived longer than 3 months. Figure 1 shows the scattergraph of M-protein decrement versus survival for this patient group. The analyses showed that these six variables contributed only 20% of the variance of the observed survival, with percentage M-protein decrement (15%) and creatinine (4%) being the major factors. Again the conclusion reached is that the clinical predictive value of these factors is minimal.

The percentage M-protein decrement was treated as a continuous variable in the above analysis while it was treated as a discontinuous variable by Alexanian *et al.* (1972) in the often quoted study. Consequently, we analysed our data in a similar fashion to Alexanian *et al.*, with four classes of serum M-protein decrement, namely, 0–50%, 51–75%, 76–90% and greater than 90%. The survival curves for myeloma patients falling into these four classes and who survived longer than 3 months are shown in Figure 2. Log rank analysis showed that these survival curves were not statistically different ($P=0.16$). Furthermore, trend analysis of these survival curves showed that a statistically significant trend was also not evident ($P=0.07$). The median survivals for these four classes was 26, 32, 46, and 39 months, respectively. The comparable values from the earlier study (Alexanian *et al.*, 1972) were 13, 19, 31 and 37 months respectively.

**Table I** Single factor analysis for the prognostic effect of various factors of survival (analysis of the 124 patients who survived longer than 3 months)

| *Variable* | P-*value* | R'-*statistic* |
|---|---|---|
| % M-protein decrement | 0.069 | 0.041 |
| Creatinine | 0.0003 | 0.10 |
| Haemoglobin | 0.038 | 0.040 |
| Calcium | 0.28 | 0.00 |
| Ig level at diagnosis | 0.24 | 0.00 |
| Age | 0.55 | 0.00 |

**Table II** Multivariate analysis using the Cox proportional hazards model for the prognostic effect of various factors on survival (analysis of the 124 patients who survived longer than 3 months)

| *Variable* | P-*value* | R'-*statistic* |
|---|---|---|
| % M-protein decrement | 0.011 | −0.077 |
| Creatinine | 0.0022 | 0.099 |
| Haemoglobin | 0.049 | −0.050 |

Model $\chi^2=22.92$ with 3 d.f. (score stat.), $P=0.0001$, *R*-statistic$=0.125$.

Test for all variables not in the model, residual $\chi^2=0.15$ with 3 d.f., $P=0.98$.

**Figure 1** Scattergraph of survival (in months) by percentage reduction of M-protein in deceased patients surviving greater than 3 months.

**Figure 2** Actuarial survival curves of patients surviving greater than 3 months. The percentage serum M-protein reduction for the four groups was 0–50% (△); 51–74% (●); 75–90% (□); >90% (○).

### Discussion

While the relationship between survival and response, which is based largely on serum M-protein decrement, is a central feature of the myeloma literature, there is surprisingly little data available which substantiates a strong correlation between M-protein decrement and survival. The data presented in this report is to our knowledge the first study of the relationship between percentage M-protein decrement, expressed as a continuous variable, and survival in a large myeloma patient population. Our data shows that while there is a statistically significant relationship between percentage M-protein decrement and survival, the predictive value of this parameter is negligible in light of the very low values of the $R'$-statistic. The linear regression analysis of data from deceased patients showed that only 15% of the variance of the observed survival could be attributed to percentage M-protein decrement. It is important to stress that it is the predictive value of a prognostic factor, as reflected in the value of the correlation coefficient, and not merely the $P$-value that determines its clinical relevance. In our opinion this latter point has been almost totally ignored in assessing the importance of prognostic factors in multiple myeloma.

The most often cited reference given in support of the relationship between serum M-protein decrement and survival is the 1972 report of Alexanian *et al.*, in which these authors concluded that a reduction of 75% or more in the rate of M-protein production resulted in an increased median survival over those patients who did not attain this level of M-protein decrement (Alexanian *et al.*, 1972). This conclusion was based on a statistical comparison of median survivals of four groups of patients who had attained differing degrees of M-protein decrement. No data were presented on the survival curves of these four groups. The paired comparisons of components of a four class analysis which was reported in this study is not currently accepted as being statistically valid in the absence of data showing that the four classes as a whole are statistically different. Furthermore, the fact that the patient population studied in this earlier report had been treated with two different drug regimens during different time periods, further complicating the interpretation of the data. Analysis of survival curves from our patient population, stratified in a similar manner to that of Alexanian *et al.* (1972), failed to show a significant difference by log rank analysis or trend analysis. Consequently, it is our contention that the conclusion subsequently drawn by other investigators that the 1972 data firmly establish the relationship between survival and M-protein decrement is unwarranted.

The weak correlation between survival and M-protein decrement is not really surprising. For a strong correlation to exist one must hypothesise that tumour mass is related to survival and that serum M-protein concentration always reflects tumour mass. It is this latter point that is highly questionable. In order for the serum M-protein concentration to always reflect tumour mass we must assume (a) that the M-protein synthetic rate is similar in all myeloma plasma cells, (b) that this synthetic rate is independent of changes in the natural history of the individual tumour, (c) that this synthetic rate is independent of any host factors, and (d) that this synthetic rate is independent of chemotherapy and radiation. We believe these assumptions are rather unrealistic in the light of what is currently known about the many factors that can alter the extent of protein production by affecting the cellular rates of transcription and/or translation.

In the absence of a currently available curative treatment in myeloma, one aim of management has been to attempt to achieve disease stabilisation. It might then appear desirable to assess the prognostic significance of achieving stabilisation. However, such an analysis is confounded for the following reason. Taking the definition of disease stabilisation to be a variance in the M-protein about the mean of no more than 10% over a minimum of 4 months, the median time to achieve stabilisation was 10.1 months in a recent large NCI-Canada myeloma trial (Belch *et al.*, 1988). That is, it takes a comparatively long time to achieve stabilisation (i.e. greater than 10 months in half of all patients), which introduces an enormous guarantee time. The result of this is inevitably that if you achieve stabilisation your survival is better than if you don't or, more succinctly, if you live a long time, you live a long time. While undeniable, this does not advance our knowledge.

Whatever the cause of the weak correlation between survival and therapy-induced M-protein decrement, extreme care should be used in the importance attached to M-protein decrement. For instance, the design of future clinical trials of conventional therapy should not use response or M-protein decrement for randomisation criteria to determine further therapy, as has been done in the past (Belch *et al.*, 1988; Mandell *et al.*, 1987; Cohen *et al.*, 1986). Response as defined largely by M-protein decrement should not be used for interim assessment of clinical trials, as has been universally the case in the myeloma literature since standard response criteria were established for myeloma (Chronic Leukemia-Myeloma Task Force, 1973; Salmon *et al.*, 1982). The absence of a treatment-induced serum M-protein decrement should not be used as the sole basis for terminating existing therapy in favour of an alternative therapy. Finally, in the light of our data, it is extremely important that the effectiveness of the very aggressive and toxic first-line therapies currently being evaluated for myeloma be assessed by survival data alone. Hopefully, the M-protein decrement obtained in myeloma patients attaining complete remission with these aggressive therapies will be significantly greater than that obtained with current conventional therapies and will provide a better predictive correlation with survival than could be demonstrated in this study.

This work was supported by MRC (Canada) and the Alberta Heritage Fund: Applied Cancer Forum. M. Palmer is the recipient of an Alberta Heritage Foundation for Medical Research Clinical Fellowship.

### References

ALEXANIAN, R., BONNET, J., GEHEN, E. & 5 others (1972). Combination chemotherapy for multiple myeloma. *Cancer*, **30**, 382.

BELCH, A., SHELLEY, W., WILSON, K. & 4 others (1988). A randomised trial of maintenance versus no maintenance melphalan and prednisolone in responding multiple myeloma patients. *Br. J. Cancer*, in the press.

CHRONIC LEUKEMIA-MYELOMA TASK FORCE (1973). Guidelines for protocol studies. *Cancer Chem. Rep.*, **4**, 145.

COHEN, H.J., BARTOLUCCI, A.A., FORMAN, W.B. & SILBERMAN, H.R. (1986). Consolidation and maintenance therapy in multiple myeloma: randomised comparison of a new approach to therapy after initial response to treatment. *J. Clin. Oncol.*, **4**, 888.

COX, D.R. (1972). Regression models and lifetables. *J. R. Stat. Soc., Series B*, **34**, 187.

DURIE, B.G.M. & SALMON, S.E. (1975). A clinical staging system for multiple myeloma: correlation of measured myeloma cell mass with presenting clinical features, response to treatment and survival. *Cancer*, **36**, 842.

HORRELL, F.E. (1986). PHGLM Procedure. In *SAS Institute Inc. SUGI Supplement Library Users Guide, Version 5* p. 437. Cory NC: SAS Institute.

KAPLAN, E. & MEIER, P. (1958). Non-parametric estimation from incomplete observations. *J. Am. Stat. Assoc.*, **53**, 452.

MANDELL, F., TRIBALTO, M., CANTONETTI, M. & 7 others (1987). Recombinant alpha 2b interferon as maintenance therapy in responding multiple myeloma patients (abstract). *Blood*, **70**, Suppl. 1, 247a.

PALMER, M., BELCH, A., BROX, L., POLLOCK, E. & KOCH, M. (1987). Are thhe current criteria for response useful in the management of multiple myeloma? *J. Clin. Oncol.*, **5**, 1373.

PETO, R. & PETO, J. (1972). Asymptotically efficient invariant procedures, *J. R. Stat. Soc., Series A*, **135**, 185.

SALMON, S.E., ALEXANIAN, R. & DIXON, D. (1982). Chemoimmunotherapy for multiple myeloma: Effect of levamisole during maintenance: Preliminary report of a Southwest Oncology Group Study. In *Immunotherapy of Human Cancer*, Ferry, W.D. & Rosenberg, S.A. (eds) pp. 61–66. Elsevier: New York.

WOCHNER, R.D., STROBER, W. & WALDMANN, T.A. (1967). The role of the kidney in the catabolism of Bence Jones proteins and immunoglobulin fragments. *J. Exp. Med.*, **126**, 207.